# microRNA expression in acute myeloid leukaemia: New targets for therapy?

**DOI:** 10.1002/jha2.441

**Published:** 2022-04-26

**Authors:** Daniel Fletcher, Elliott Brown, Julliah Javadala, Pinar Uysal‐Onganer, Barbara‐ann Guinn

**Affiliations:** ^1^ Department of Biomedical Sciences University of Hull Hull, UK; ^2^ Cancer Research Group School of Life Sciences University of Westminster London UK

**Keywords:** acute myeloid leukaemia (AML), microRNA, miRNA, stemness, therapy

## Abstract

Recent studies have shown that short non‐coding RNAs, known as microRNAs (miRNAs) and their dysregulation, are implicated in the pathogenesis of acute myeloid leukaemia (AML). This is due to their role in the control of gene expression in a variety of molecular pathways. Therapies involving miRNA suppression and replacement have been developed. The normalisation of expression and the subsequent impact on AML cells have been investigated for some miRNAs, demonstrating their potential to act as therapeutic targets. Focussing on miRs with therapeutic potential, we have reviewed those that have a significant impact on the aberrant biological processes associated with AML, and crucially, impact leukaemic stem cell survival. We describe six miRNAs in preclinical trials (miR‐21, miR‐29b, miR‐126, miR‐181a, miR‐223 and miR‐196b) and two miRNAs that are in clinical trials (miR‐29 and miR‐155). However none have been used to treat AML patients and greater efforts are needed to develop miRNA therapies that could benefit AML patients in the future.

**Abbreviations**
AKT: Alpha serine/threonine kinase
AML: Acute myeloid leukaemia
BCL11B: B‐cell lymphoma/leukaemia 11B
CAF1: Chromatin assembly factor 1
CAMKK1: Calcium/calmodulin‐dependent protein kinase kinase 1
CDK6: Cyclin‐dependent kinase 6
CFU: Colony forming unit
CLL: Chronic lymphocytic leukaemia
CR: complete remission
CTDSPL: Small phosphatase‐like
DNMT: DNA methyltransferase
DS: Down syndrome
E2F1: E2F transcription factor 1
EiF4G: Eukaryotic translation initiation factor 4G
ETO: RUNX1 transcriptional co‐repressor 1
EVI1: Ectopic viral integration site 1
FAB: French‐American‐British
FLT3: Fms‐related receptor tyrosine kinase 3
GATA1: GATA binding protein 1
HIF‐1A: Hypoxia inducible factor‐1
HMGB1: High mobility group box 1
HOX: Homobox cluster
HSC: Haematopoietic stem cell
ITD: Internal tandem duplication
KLF5: Krüppel‐like factor 5
LSC: Leukaemic stem cell
MCL‐1: Myeloid‐cell leukaemia‐1
MDM2: Mouse double minute 2
MDR1: Multi‐drug resistance protein 1
MEIS1: Meis homobox 1
miRNA: MicroRNA
MLL: Mixed lineage leukaemia
Tf‐NP: Transferrin‐conjugated lipid nanoparticle nanoparticle
NPM1: Nucleophosmin 1
PDCD4: Programmed cell death 4
PI3K: Phosphoinositide 3‐kinase
PRKCD: Protein kinase C delta
PTEN: Phosphatase and tensin homolog
RER: Rough endoplasmic reticulum
SHIP1: SH2 domain‐containing inositol 5’‐phosphate 1 protein
TET1: Ten‐eleven translocation methylcytosine deoxygenase 1
VEGFA: Vascular endothelial growth factor A
XRN1: 5‐3 exoribonuclease 1.

## 1 INTRODUCTION

The treatment methods that are currently available for adults with acute myeloid leukaemia (AML), commonly achieve remission, but are often followed by relapse and a poor prognosis. SEER (https://seer.cancer.gov/statfacts/html/amyl.html) estimates that the average 5‐year overall survival (OS) in AML is 29.5%, a 6.1% increase over the previous decade and a 12.5% increase over Rowe's study [1]. The differences in survival rates reflect significant differences in the underpinning AML biology and in patient responses to treatments [2]. It also highlights patient demographics as a factor for disease outcome, with the traditional 7+3 regimen being especially difficult to tolerate for some older or frail patients. These patients experience less toxicity if treated with non‐intensive regimens but also have a lower probability of achieving complete remission (CR) [3, 4].

Many of the biological therapies used to treat AML are inhibitory and block the aberrant gene product, cell cycle/proliferation and epigenetic regulators implicated in AML, such as the Fms‐related receptor tyrosine kinase 3 (FLT3) inhibitor, Rydapt or the Bcl‐2 inhibitor, venetoclax [5, 6]. Recent advances in medicine herald a new era for genetic and epigenetic therapies and the opportunity to treat the genetic cause of disease rather than treating the product of a gene mutation [7]. While CRISPR/Cas9 gene editing solutions have shown success in minimising the formation of leukaemic stem cell (LSC) colonies, these therapies remain in the earliest stages of clinical application [8].

Recent studies have shown that microRNAs (miRNAs) and their dysregulation are implicated in the pathogenesis of AML due to their role in the control of gene expression that impacts on a variety of molecular pathways [9]. Therefore miRNAs represent a new therapeutic opportunity that can treat the cause of disease at the transcriptional level and unlike CRISPR/Cas9, miR therapy is unlikely to cause off‐targets effects due to their specificity for a seed sequence [10].

### 1.1 miRNAs

miRNAs are short non‐coding RNAs of approximately 20–25 nucleotides in length. Around 50% of miRNAs are intragenic, predominately transcribed from intronic sequences, while the remaining miRNAs are intergenic and subject to their own promoters, independent of host gene transcription [11]. miRNAs are pivotal in gene regulation through their gene silencing activity which causes mRNA decay, either through the formation of a complex or via interactions with specific proteins. Conversely miRNAs are implicated in translation activation and the upregulation of mRNA production [12, 13].

**FIGURE 1:**
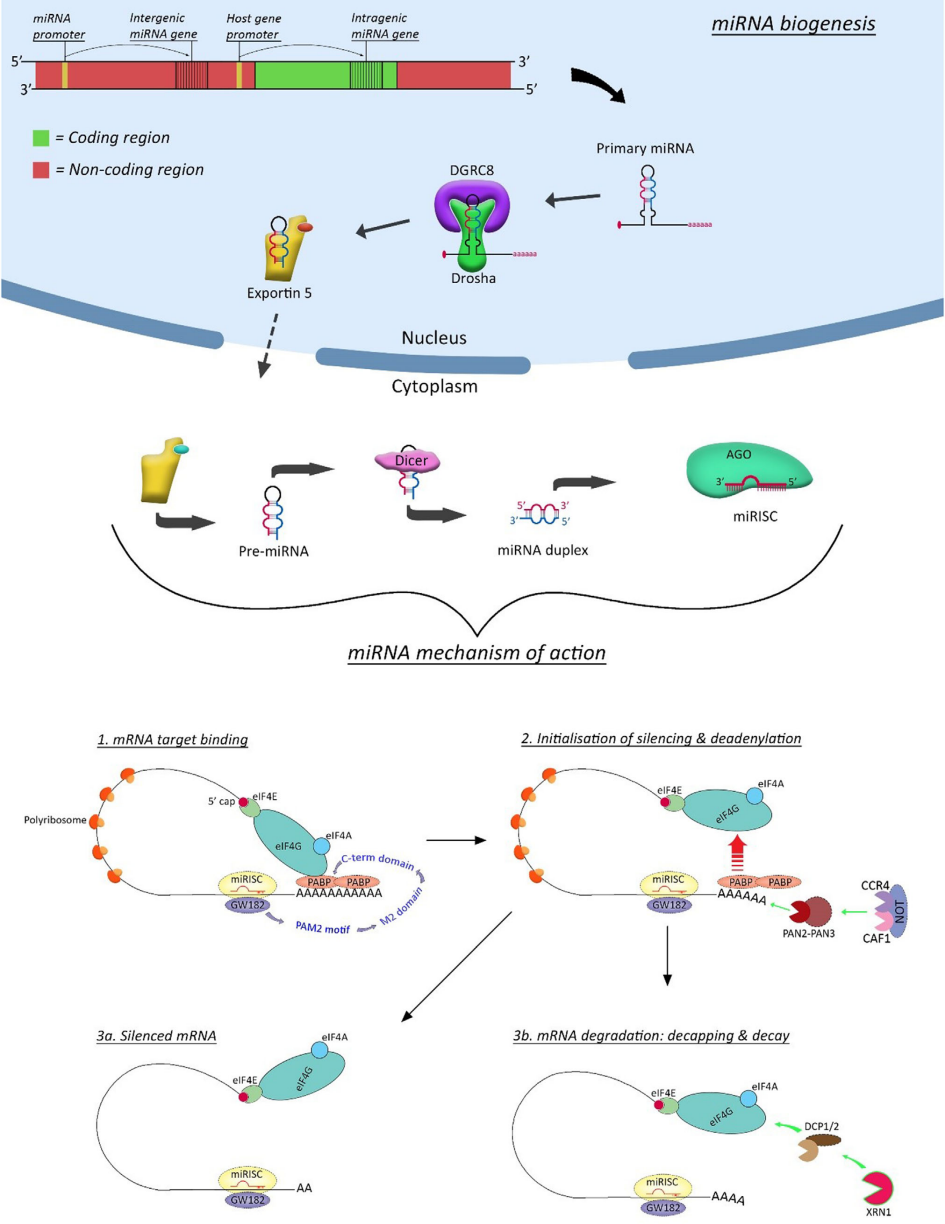
**microRNA biogenesis**. miRNA synthesis within the nucleus from intragenic genes is subject to host‐gene promoter regulation. Intergenic miRNA genes, those contained within non‐coding regions, are subject to their own promoters and transcription is independent of host‐genes. DGCR8 and Drosha recognition of primary‐RNA, via DGCR8/N6‐mth‐GGAC motif recognition, processes the pri‐miRNA and cleaves the hairpin structure, and leaves a 3’ overhang. The pre‐miRNA is transported to the cytoplasm by Exportin 5/RanGTP. The endonuclease Dicer cleaves the pre‐miRNA terminal loop to produce the mature miRNA duplex. The two strands of the mature duplex are 3p and 5p and only one strand is designated the guide strand and loaded into the AGO family protein to form the miRISC. The ratio of 5p and 3p loaded into AGO varies. **(A)** The ‘seed’ region (approx. 2–8 nt) in the 5’ end of the mature miRNA complementarily binds to the 3’ UTR of the target mRNA and AGO interacts with GW182 family protein. GW182 itneracts with PABP via PAM2 and M2 motifs. **(B)** Interaction of GW182 and PABP competitively inhibits interaction of PABP with eIF4 proteins, destabilising the mRNA loop structure and initiating deadenylation. PAN2/3 complex reduces the poly‐A tail to approx. 50–110 nt and the CAF1‐CCR4‐NOT complex degrades the remainder of the poly‐A tail. **(Ci)** Upon completion of deadenylation, the mRNA/miRISC can be maintained in a silenced mRNA state; **(Cii)** DCP1/2 decaps the deadenylated target mRNA and the exonuclease XRN1 completes mRNA decay with 5’‐3’ degradation

While the canonical and non‐canonical miRNA biogenesis pathways differ within the nucleus (Figure 1), once miRNAs have been transported to the cytoplasm by Exportin5/RanGTP, the mature miRNA complexes with Argonaut (AGO) family proteins to form the miRNA‐induced silencing complex (miRISC) [11]. A ‘seed’ sequence at the 5’ end of the miRNA binds to a complimentary sequence in the 3’UTR of the target mRNA and AGO interacts with GW182 family proteins. GW182 binds to the poly‐A‐binding protein and competitively inhibits its interaction with eukaryotic translation‐initiation factor 4G (eIF4G), destabilising the closed loop structure of the mRNA and initiating deadenylation [14]. A miRISC/GW182 complex can remain in situ and form a silenced mRNA or the GW182 protein can recruit the decapping protein 1/2 to remove the mRNA 5’ cap and proceed to complete mRNA degradation by the 5‐3 exoribonuclease 1 (XRN1) [12, 14].

miRNAs have numerous subcellular locations including the nucleus, the initiation site of miRNA biogenesis (Figure 1), but are also found in the cell cytoplasm of endosomes, exosomes, lysosomes, P‐bodies, mitochondria, the Golgi network and the rough endoplasmic reticulum (RER) in addition to the circulatory system [11]. The RER membrane is rich in miRNA target messages and is a key site of de novo mRNA translation repression [15]. P‐bodies form as a consequence of mRNA deadenylation and as such are rich in miRNA, GW182 proteins, decapping proteins and the CAF1‐CCR4‐NOT complex.

While miRNAs and their targets have numerous regulatory roles and functions under normal physiological conditions (Table 1), for a target to have therapeutic potential it needs to also have a functional effect in the disease. To this end some miRNAs have been shown to play a role in key features in leukaemia including stemness and therapy resistance.

**TABLE 1:** Summary of miRNA's identified and their function under normal physiological conditions

miRNA	Normal physiological function	References
miR‐9	**Development**: Regulates neural progenitors – differentiation and proliferation. **Immune**: Implicated in LPS‐induced inflammatory response	[79, 80]
miR‐21	**Immune**: T‐cell activation and maintenance. Potential regulation of Th1‐cell immune response. **Development**: Regulation of self‐renewal. Promotion of branching morphogenesis.	[81]
miR‐29	**Immune**: T‐cell differentiation and activation. B‐cell proliferation and antibody generation. **Myogenesis**: Atrophy and differentiation of muscle cells. **Osteogenesis**: Osteoblast differentiation. Regulation of osteoclast production. **Haematopoiesis**: Stimulation of HSPC and HSC self‐renewal. **Tumour suppressor**: Regulates apoptosis.	[82]
miR‐34	**Tumour suppressor**: Regulates differentiation, apoptosis, cell senescence, cell cycle arrest, apoptosis and self‐renewal.	[83]
miR‐125	**Immune**: Implicated in host defence via IFN and TNF. Interruption of viral translation. Inflammation regulation via NF‐κβ. T‐cell activation. **Haematopoiesis**: Regulation of survival, proliferation and differentiation. Putative regulation of HSC homeostasis.	[44, 84]
miR‐126	**Haematopoiesis**: Regulation of HSPC proliferation and differentiation. Modulates endothelial cell phenotype. Regulates vascular integrity. Regulates angiogenesis.	[19, 85]
miR‐139	**Tumour suppressor**: regulates survival, proliferation and differentiation in many malignancies.	[86, 87]
miR‐142	**Haematopoiesis**: Regulation of erythropoiesis and megakaryopoiesis. **Immune**: Regulates activated T‐cell cycle. Enhances activity of mast cells. Regulates dendritic cell homeostasis.	[88, 89]
miR‐155	**Haematopoiesis**: HSC maturation – myelopoiesis and erythropoiesis. **Immune**: Regulates B‐cell antibody‐mediated signalling. Regulates T‐cell host defence and inflammatory response. Regulation of macrophage and dendritic cell cytokine production.	[90, 91]
miR‐181	**Development**: Regulates embryo implantation. Regulates CNS cell differentiation – neurons, ganglion cells, retinal cells. **Immune**: Regulates HSPC differentiation – granulocyte and macrophage‐like. Regulation of cell cycle progression.	[66, 92]
miR‐196	**Development**: Implicated in embryogenesis via HOX family genes. Regulation of differentiation, apoptosis and proliferation.	[93]
miR‐199	**Tumour suppressor**: Via K‐RAS and Akt. **Development**: Regulates proliferation, differentiation and migration in cardiac, muscular, reproductive and respiratory systems.	[94]
miR‐210	**Haematopoiesis**: Putative role in erythropoiesis. Inhibits proliferation and cell cycle progression, promotes differentiation, and regulates apoptosis. Hypoxia: Regulates mitochondrial metabolism and DNA repair under oxidative stress. Increases stem cell survival in hypoxic environments.	[95]
miR‐223	**Immune**: Regulates granulocyte and dendritic cell differentiation. Polarization of macrophages in immune/inflammatory response. Regulates proliferation and differentiation of T cells. Implicated in T‐cell inflammatory response. Modulates endothelial and epithelial inflammation. **Thrombopoiesis**: putative role in platelet reactivity.	[96, 97]
miR‐378	**Metabolism**: Involved in lipid metabolism, gluconeogenesis and adipogenesis. Regulates cytochrome P450. Regulates mitochondrial function. Implicated in differentiation and regeneration of muscle cells including cardiomyocytes. Regulates angiogenesis via VEGFA. **Immune**: Regulates NK‐cell cytotoxicity and macrophage inflammatory response.	[98]
miR‐451	**Haematopoiesis**: Regulation of erythrocyte maturation. Inhibits erythrocyte apoptosis under hypoxic conditions. Associated with resistance to Malaria.	[99]
miR‐486	**Haematopoiesis** : Regulates differentiation, proliferation and survival of erythroid cells.	[100]

### 1.2 miRNAs and stemness

Stemness of leukaemic cells is a major contributor to AML leukaemogenesis and LSCs play a significant role in disease relapse [16]. Recent studies have highlighted the role of mir‐125 in leukaemogenesis and haematopoietic stem cell (HSC) self‐renewal in Mixed Lineage Leukaemia (MLL)‐translocated AML, whereby miR‐125a overexpression upregulated vascular endothelial growth factor A (VEGFA) and was associated with a significantly reduced median survival in MLL/miR‐125 transduced mice. Secondary AML caused by doxycycline‐induced miR‐125 overexpression was reversed by the inhibition of miR‐125b resulting in improved OS and event free survival [17, 18].

miR‐126 was also active in HSC regulation and further involved in LSC status in AML. Lechman and Gentner [19] discovered a 40‐fold increase of miR‐126 in LSC containing populations and the overexpression of miR‐126 induced an increase in quiescent primitive cells (CD34^+^) and decreased the population of differentiated CD14^+^CD15^+^ cells. The miR‐126^high^ LSCs also displayed greater resistance to certain chemotherapeutic agents. The knockdown of miR‐126 demonstrated the capacity to direct primitive CD34^+^ cells into a more committed state. In Homobox (HOX)‐driven AMLs, miR‐21 and miR‐196 were shown to be competitively regulated by GFI1 and HOXA9 and jointly upregulated in 11q23 AML where they are implicated in the formation of leukaemia initiating cells [20].

### 1.3 miRNAs and therapy resistance

Therapy resistance is a major hurdle in the pathogenesis of AML, with numerous mechanisms, including LSCs, attributed to chemoresistance [21, 22]. miR‐21 has been observed to work with other miRNAs in AML such as miR‐15a to cause chemoresistance. Both miR‐21 and miR‐15a were found to downregulate programmed cell death 4 (PDCD4), BTG antiproliferation factor 2 (BTG2) and ADP ribosylation factor‐like protein 2 (ARL2) and reduce chemotherapy‐induced apoptosis [23]. miR‐181b was found to inhibit high mobility group box 1 (HMGB1) and myeloid leukaemia (MCL‐1) while HMGB1 was expressed at high levels in relapsed/refractory AML patients. Suppression of HMGB1 via RNA interference sensitized multidrug‐resistant leukaemia cells to chemotherapy and induced apoptosis [24]. The downregulation of miR‐451 was responsible for the increase in multidrug resistance protein 1 (MDR1) in FLT3‐internal tandem duplication+ (ITD) AML, and as such contributed to the poor therapeutic response [25] seen in these patients.

### 1.4 miRNA targeting for cancer therapy

The clinical significance of miRNAs as targets for cancer therapy was highlighted by [26] who discovered that a small deletion in chromosome 13q14 in chronic lymphocytic leukaemia (CLL) resulted in a loss or downregulation of two miRNAs, miR‐15a and miR‐16‐1, both of which target multiple known oncogenes [27]. Further investigation showed that miRNA genes commonly reside in genomic regions implicated in cancer and as such, are often deleted or over/underexpressed as a consequence of their close proximity to oncogenes.

miRNA therapies involving their suppression or replacement have been developed [28]. miRNA suppression therapies involve the binding of synthetic miRNAs to host miRNA or target mRNA in diseases where miRNA upregulation is implicated. Conversely, miRNA replacement therapy involves the integration of synthetic miRNA into target cells/tissues in disease where miRNA deletion or downregulation has occurred [29].

### 1.5 miR targets for therapy in AML – overexpressed miRs

**FIGURE 2:**
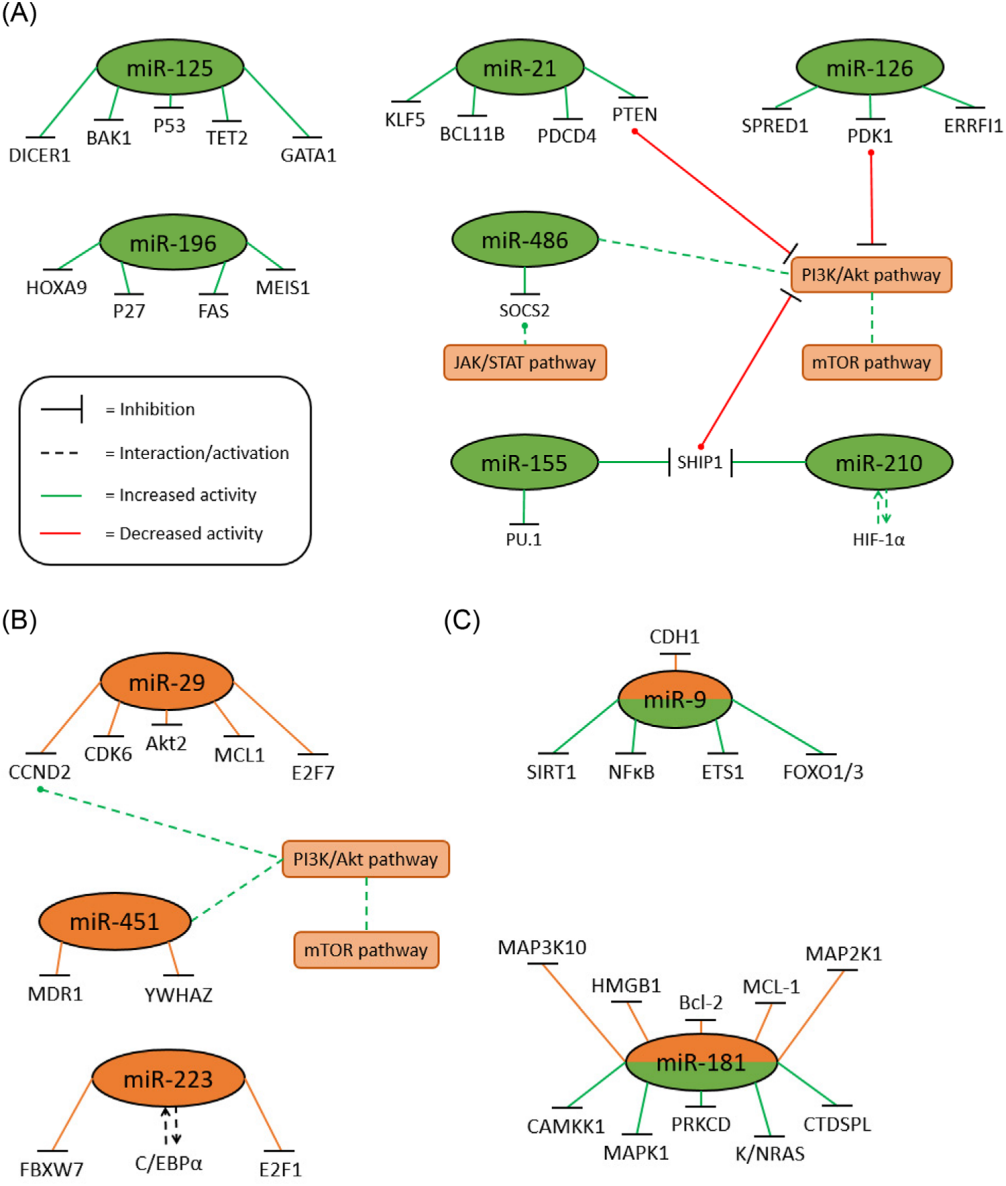
**Subversions of miRNAs in AML**. microRNAs are (**A**) upregulated, (**B**) downregulated or (**C**) dysregulated. Many of these miRNAs directly or indirectly target common protein families such as cyclins and cyclin‐dependant kinases, or common pathways such as the PI3K and MAPK pathway. These microRNAs are key components of haematopoiesis and their preferential targets regulate key aspects of the cell cycle, maturation and cellular senescence, and as such their dysregulation is implicated in leukaemogenesis. Secondary downstream targets and pathways in black

The potential of miRs to act as targets for therapy are based on their subversion. For example miR‐21 is a prominent miRNA in a range of malignancies including oral squamous carcinoma and glioma, and in AML it provides an example of the complexity of targeting mRNA regulation [30, 31, 32] (Figure 2A). miR‐21 is overexpressed in all French‐American‐British (FAB) subtypes of AML, with greater prevalence in M3, M4, M5 and NPM1‐mutated AML [33, 34] (Table 2). A number of its targets are drivers of leukaemogenesis, including B‐cell lymphoma/leukaemia 11B (BCL11B), and Zhang et al. [35] demonstrated increased proliferation, reduced apoptosis and inhibited differentiation via BCL11B‐mouse double minute 2 (MDM2) in Thp‐1 cells overexpressing miR‐21. Targeting of the tumour suppressors PDCD4 and phosphatase and tensin homolog (PTEN) by miR‐21 was established by Riccioni et al. [34] as playing key roles in differentiation arrest and reduced apoptosis in NPM1 mutated AML. Both studies commented on the immature natural killer‐cell phenotype created by miR‐21 overexpression. Further elucidation of miR‐21 as an oncomiR was provided by Li et al. [36] who established miR‐21 functions via another of its targets, Krüppel like factor 5 (KLF5), to drive a proliferative state in AML and even operate outside of the myeloid lineage. Moussa Agha et al. [37] discovered miR‐21 negatively impacts T‐cell fragility and potentially disrupts innate tumour defence mechanisms. miR‐21 was found to be underexpressed in cytogenetically normal AML with high Tet methylecytosine deoxygenase 1 (TET1) and while some studies have found miR‐21 to target TET1 in colorectal cancer, it was TET1 overexpression, and not miR‐21 underexpression, that was suggested as the contributor to AML progression in this instance [38].

**TABLE 2:** Summary of miRNAs identified as potential therapeutic targets in AML

miRNA	Association with subtype/genetic abnormalities	Targets/pathways	References
miR‐9	MLL‐AML, EVL1‐ETO t(8;21), Paediatric AML	CCNG1, CDH1, CDX2, ETS‐1, FOXO1/3, ID2, SIRT1, STMN1, NFκB	[65], [101], [64]
miR‐21	NPM1, all FAB subtypes	BCL11B, KLF5, MDM2, PDCD4, PTEN	[37], [36], [34], [35]
miR‐29		AKT2, CCND2, CDK6, DNMT, E2F7, MCL‐1	[75], [54]
miR‐34	CEBPA mutation	E2F3, HMGB1, SIRT1	[102], [103]
miR‐125	AML1‐ETO(FLT3+), MLL‐AF9, APML (PML‐RARα), DS‐AML, t(2;11), t(11;14)	DICER1, NKIRAS2 (KBRAS2), TP53, TET2, TNFα, VEGFA	[45], [17], [44]
miR‐126		CDK3, CDH7, JAG1, PDK1, PI3K‐AKT‐MTOR	[19]
miR‐139	FLT3+, NPM1	CDK6, eIF4G2, P27, PI3K‐AKT, TSPAN3	[104], [105], [106], [107]
miR‐142	IDH mutation	ASH1I, CTNNB1, HOXA	[108], [109]
miR‐155	FLT3+, M4, M5	PU.1, PI3K‐AKT, SHIP1	[110], [40]
miR‐181	M1, M2, M3, CEBPA mutation	CAMKK1, CTDSPL, HMGB1, KRAS, MCL‐1, MAPK1, NRAS, PRKCD‐P38‐C/EBPα	[111], [24], [112], [66]
miR‐196	MLL‐AML (HOX), FLT3+, M4, M5	ANXA1, ERG, FAS, HOXA9, MEIS1	[51], [113]
miR‐199	NPM1, M5	DRAM1, ERK, HIF‐1α, HOXA7, HOXB6, KRAS‐AKT	[114], [115]
miR‐210	Myelodysplastic syndrome	C/EBPα, HIF‐1α, SHIP1	[42], [43]
miR‐223	All FAB subtypes	E2F1, FBXW7, TP53	[116], [117], [57], [59]
miR‐378	AML1‐ETO, M2, t(8;21)	EPOR, FUS1, GZMB	[118], [119]
miR‐451		C‐MYC, MDR1, YWHAZ‐AKT	[62]
miR‐486	DS‐AML	PI3K‐AKT, SOCS2‐JAK‐STAT	[120], [50]

AHS1l, ASH1‐like histone lysine methyltransferase; ANXA1, annexin 1; CCND2, cyclin D2; CCNG1, cyclin G1; CDH1, cadherin 1; CDH7, cadherin 7; CDK3/6, cyclin‐dependant kinase 3/6; CDX2, caudal‐type homeobox 2; CEBPA, CCAAT enhancer binding protein 4; CTNNB1, catenin beta 1; DRAM1, DNA damage regulated autophagy modulator 1; eIF4G2, eukaryotic translation initiation factor 4 gamma 2; EPOR, erythropoietin receptor; E2F3, E2F transcription factor 3; E2F7, E2F transcription factor 7; ERK, extracellular signal‐regulated kinase; ERG, ETS transcription factor ERG; ETS‐1, ETS proto‐oncogene; FOXO1/3, forkhead box O1/O3; FUS1, tumour suppressor 1 calcium regulator; GZMB, granzyme B; ID2, inhibitor of DNA binding 2; JAG1, jagged 1; KRAS, KRAS proto‐oncogene; MAPK1, mitogen‐activated protein kinase 1; NFκB, nuclear factor kappa B; NKIRAS2, NFκB inhibitor‐interacting Ras‐like 2; NRAS, NRAS proto‐oncogene; P27, cyclin‐dependant kinase inhibitor 1B; PDK1, pyruvate dehydrogenase kinase 1; PU.1, Spi‐1 proto‐oncogene; SIRT1, Sirtuin 1; SOCS2, suppressor of cytokine signalling 2; STMN1, stathmin 1; TET2, Tet methylcytosine dioxygenase 2; TNFα, tumour necrosis factor alpha; TP53, tumour protein 53; TSPAN3, tetraspanin 3; YWHAZ, protein zeta.

The oncogenic miR‐155‐5p plays a role in haematopoiesis and cell differentiation, while overexpression has been shown to be associated with lower survival rates in CLL patients (*n* = 88; *p* = 0.001) [39]. Upregulation of miR‐155 inhibits SH2 domain‐containing inositol 5’‐phosphate 1 protein (SHIP1)‐phosphoinositide 3‐kinase (PI3K)‐alpha serine/threonine kinase (AKT) pathway and significantly reduces apoptosis in CLL and AML cells [40, 41]. miR‐210 also targets SHIP‐1, which along with miR‐155 has been associated with the loss of SHIP‐1 in high‐risk myelodysplastic syndrome patients [42]. Elevated expression of miR‐210 was observed in AML patients where it correlated with poor outcomes (overall and event free survival) and was significantly lower in patients achieving CR. Hypoxia inducible factor‐1α (HIF‐1α) enhances miR‐210 which in turn can stabilise HIF‐1α. This positive feedback loop could be a factor in miR‐210 overexpression given the hypoxic bone marrow microenvironment in AML [43].

The miR‐125 family comprising miR‐125b1, miR‐125b2 and miR‐125a is located on chromosome 11, 21 and 19, respectively, as part of conserved clusters with miR‐100 and paralogs of miR‐99 and Let‐7. The miR‐125a cluster has a single promoter and is transcribed as one, whereas miR‐125b contains an additional promoter which enables the transcription of miR‐125b on its own [44]. An investigation of AML with t(2;11) in a murine model showed up to a 90‐fold increase in expression of miR‐125b, with miR‐125b‐transplanted mice developing myeloproliferative disorders that progressed to AML. It was noted that differing levels of miR‐125b correlated with different leukaemic phenotypes, suggesting that the level of overexpression not only contributes to the disease state but also to the disease phenotype [45, 46].

miR‐125b2 is located on chromosome 21 and overexpressed in Down's syndrome (DS) patients with acute megakaryocytic leukaemia. GATA binding protein 1 (GATA1) mutations that result in truncated GATA1s are key drivers of leukaemogenesis in DS associated‐AML and Klusmann et al. [47] determined that GATA1s‐induced proliferation, self‐renewal and megakaryocyte colony forming unit (CFU) numbers were more aggressive in the presence of miR‐125b2. Interestingly, the study discovered DICER1, integral to miRNA biogenesis and miRISC assembly, was a direct target of miR‐125b2, suggesting that miR‐125b2 may harbour the potential to disrupt/dysregulate overall miRNA synthesis.

During our own analysis of differential gene expression between risk subgroups in AML, miR‐486 was found to be differentially expressed between intermediate versus good, and poor versus good risk subgroups of adult AML patients [48] and elevated in expression in high risk AML patients [49]. As detailed by Shaham et al. [50], miR‐486 was also overexpressed in DS‐AML and had the same synergy with GATA1s as miR‐125b2, although with less acute effects on leukaemogenesis. Unlike miR‐125b2, GATA1 appears to enhance the expression of miR‐486 as a result of its intronic location in the ankyrin 1 gene.

miR‐196 highlighted another facet of miRNA regulation complexity in AML. The forced expression of miR‐196b inhibited oncogenes HOXA9 and Meis homobox 1 (MEIS1) and delayed leukaemogenesis. In contrast, the same study showed that miR‐196b inhibited the tumour suppressor Fas cell surface death receptor (FAS) and was linked to reduced apoptosis, increased proliferation and repressed differentiation [51]. There was also an investigation of miR‐196b in paediatric AML where miR‐196b expression was highest in FAB M4/5 and FLT3+ITD AML, and specifically correlated with poor remission rates after initial induction and overall poor prognosis [52].

### 1.6 miR targets for AML therapy – underexpressed miRs

Underexpressed miRs (Figure 2B) provide an opportunity for therapeutic replacement and return of the lost contribution of specific miRs as controllers of gene expression. For example, a number of miR‐29 family targets are found in malignancies such as DNA methyltransferases (DNMT) in lung cancer and histone deacetylases [53]. miR‐29 is also linked to oncometabolism, with miR‐29 downregulation and PI3K‐Akt de‐inhibition promoting tumour growth in ovarian cancer [54]. Initial studies on miR‐29 underexpression in AML showed that it targets protein kinase b (Akt2) and cyclin‐D2 protein, along with a negative feed‐back loop of MYC proto‐oncogene (c‐Myc)‐Akt2 onmiR‐29. This loop was implicated in leukaemogenesis and the introduction of miR‐29 back into bone marrow blasts partially restored normal apoptotic activity and myeloid cell differentiation [55].

One of the most prominent miRNAs involved in granulopoiesis, miR‐223, was consistently downregulated in various AML subtypes including those patients with C/EBPα and AML1 mutations. The AML1/RUNX1 transcriptional co‐repressor 1 (ETO) fusion protein silences miR‐223 and inhibits cell differentiation [56]. C/EBPα is a known regulator of miR‐223 and commonly mutated in AML. The mutated C/EBPα suppresses miR‐223 and forms a negative feedback loop whereby overexpression of the miR‐223 target E2F transcription factor 1 (E2F1) suppresses miR‐223. E2F1 is a major regulator of cell cycle progression, its disinhibition as a result of miR‐223 silencing increases proliferation and disrupts cell cycle progression [57]. miR‐223 underexpression also leads to overexpression of another of its targets, F‐box and WD repeat domain containing 7 protein (FBXW7), which was demonstrated as contributing to proliferation and reduced apoptosis in AML [58]. A recent study determined that low miR‐223 was a significant independent indicator of poor OS in AML patients while an elevated expression of miR‐223 was more prevalent in patients with CR [59].

miR‐451 is involved in the late‐stage maturation of erythroid cells and is unique amongst miRNAs. Yang et al. [60] discovered that miR‐451 maturation can occur independently of Dicer, the protein responsible for the cleavage of the pre‐miRNA hairpin loop during the formation of the mature miRNA duplex. miR‐451 is downregulated in AML and despite its activity in the erythroid lineage, the re‐introduction of miR‐451 decreased the rate of cell proliferation and increased apoptotic activity in AML cell lines [61, 62]. Miragen developed MGN 4893, a miR‐451 inhibitor, as a treatment for polycythaemia vera that is being used in clinical trials [28].

### 1.7 Dysregulated miRNAs in AML – the ‘double edged sword’ of subtype expression

The varied expression patterns of miRNAs including the miR‐9 and miR‐181 families (Figure 2C) demonstrate the delicate balance in the regulatory roles of miRNAs, targeting both tumour suppressors and oncogenes, and often in a disease‐specific manner. This results in a regulatory ‘double‐edged sword’ whereby aberrant expression (increased or decreased) contributes to different leukaemic subtypes. The miR‐9 and miR‐181 molecules highlight the dichotomous role of miRNAs in haematopoiesis and their varied expression across AML subtypes. In MLL‐ AML, miR‐9 is overexpressed as a result of direct targeting by MLL‐fusion genes. Induction of a more aggressive phenotype is attributed to miR‐9 targeting of tumour suppressors, with the inhibition of miR‐9 significantly increasing apoptosis in MLL‐cells [63]. In contrast, miR‐9 was downregulated in ectopic viral integration site 1 (EVI1)^high^ AML compared to normal and EVI1^low^ cells, and forced expression of miR‐9 by 5‐azacytidine correlated with reduced colony formation and increased apoptosis [64]. In paediatric AML with t(8;21), miR‐9 was suggested to act as a tumour suppressor, its underexpression facilitating leukaemogenesis and differentiation arrest via its targets, Lin‐28 homolog B (LIN28B) and high mobility group AT‐hook 2 (HMGA2). Reintroduction of miR‐9 was able to reverse the leukaemic effects via growth reduction and differentiation initiation without affecting apoptosis [65].

miR‐21 was found to be underexpressed in cytogenetically normal AML with high Tet methylecytosine deoxygenase 1 (TET1), while some studies have found miR‐21 to target TET1 in colorectal cancer. It was TET1 overexpression and not miR‐21 underexpression that was suggested as the contributor to AML progression in this study [38].

The miR‐181 family are overexpressed in AML M1, M2 and M3 where downregulation of protein kinase C delta (PRKCD)‐p38‐CCAAT enhancer binding protein alpha (C/EBPα) contributed to proliferation and myeloid differentiation arrest. MiR‐181a also directly targeted calcium/calmodulin‐dependant protein kinase kinase 1 (CAMKK1) and tumour suppressor CTD small phosphatase‐like protein (CTDSPL), both of which are implicated in granulocyte and macrophage‐like differentiation [66]. Conversely, downregulation of the miR‐181 family members were identified in a specific subset of cytogenetically normal AML patients and moreover, the upregulation of miR‐181 family was associated with better outcomes in C/EBPα ‐mutated AML, where the mutated C/EBPα directly targets miR‐181 [67]. Reintroduction of miR‐181b into MDR AML cells treated with doxorubicin or cytarabine significantly reduced cell growth and increased apoptosis.

### 1.8 Therapeutic potential of miRs in AML

As highlighted above, miRNAs have complex and differing expression patterns, and a variable impact on leukaemogenesis and the prognosis of AML (Table 2). The normalisation of expression to housekeeping genes such as 18S ribosomal subunit or RNU44 snoRNA, and the subsequent impact of miRNA expression in AML has been investigated using qPCR, elucidating their potential as therapeutic targets (Table 3). Of the five pre‐clinical studies (detailed in Table 3), four utilised both in vivo and in vitro models while one used an in vivo model only. All of the studies reported efficient delivery and uptake of the miR therapies, as demonstrated by relevant changes in miRNA concentration and where analysed, changes in known target protein concentrations. The miR therapies investigated had a significant impact on aberrant biological processes associated with AML, and crucially, impacts on the aforementioned hurdles in AML‐LSCs, self‐renewal capacity and therapy resistance. Furthermore, two of the studies reported no impact on bone marrow pathology or normal HSCs and no change in major organ function or pathology post‐miR therapy.

**TABLE 3:** Summary of miRNA normalisation and potential miRNA therapies

miRNA therapy	Delivery	Results	References
miR‐29b mimic	Transferrin‐conjugated lipid nanoparticle (Tf‐NP)	Reduced CFU growth rate: 6.9% in Kasumi‐1 cells, 9.1% in MV4‐11 cells and 13.6% in OCI‐AML3 cells (*p* ≤ 0.05). Increased OS by 5.5 days and 10 days – two in vivo trials of MV4‐11 transplanted mice (*p* ≤ 0.05). As a pre‐treatment with Decitabine: reduced cell viability by 40% and increased OS by 10 days (*p* ≤ 0.01).	[75]
miR‐126 antagomiR	Tf or CD45.2‐conjugated lipid NP	Significantly reduced CFUs and self‐renewal in secondary, tertiary and quaternary re‐platings (*p* ≤ 0.001, *p* ≤ 0.01, *p* ≤ 0.05). Increased OS by 15 days and 25 days respectively in mice transplanted with 10^6^ cells and 10^5^ cells.	[121]
miR‐181a mimic	Tf‐NP	>50% reduction in CFUs. Reduced proliferation: 40% in KG1a cells, 32% in MV4‐11 cells and 25% in OCI‐AML3 cells (*p* ≤ 0.05). Increased apoptosis: 13% in MV4‐11 cells and 12% in OCI‐AML3 cells. Normal pathology of bone marrow, sternum, spleen and liver in MV4‐11 engrafted mice treated with miR‐181a.	[122]
miR‐223	Lentiviral transfection	Increased the number of cells in G0/G1 and reduced the number of S‐phase cells.	[57]
miR‐21 and miR‐196b antagomiRs	AntagomiR	Curative in MLL‐AF9 transplanted mice. Increased survival in conjunction with chemotherapy induction regime. Reduced CFUs, self‐renewal and LSC maintenance (LSC free at day 150). No impact on normal behaviour or organ function, number or type of HSCs, WBCs or lymphocytes.	[20]

### 1.9 Clinical trials

Of the miRNAs reviewed here, only two are currently in clinical trials. Cobomarsen (MRG‐106) is a miR‐155 inhibitor developed by Viridian therapeutics and has demonstrated efficacy in the treatment of cutaneous T‐cell lymphoma [68]. Viridian Therapeutics also developed Remlarsen and MRG‐229, both of which are miR‐29 mimics that show potential in the treatment of tissue injury/fibrotic disease and idiopathic pulmonary fibrosis respectively [69, 70].

miR‐21, miR‐29b, miR‐126, miR‐181a, miR‐223 and miR‐196b (Table 2) have been the subjects of pre‐clinical studies and one of these was as a combination miR‐therapy (miR‐21/miR‐196b). With limited data on miRNA therapy in AML, the potential role of miR‐therapies in the treatment landscape of AML remains unclear. A number of the miRNA targets have existing treatments such as mTOR inhibitors and the pre‐clinical data from miRNA therapies demonstrates their niche impact on AML pathology. However, mTOR inhibitors such as Rapamycin have shown side‐effects including toxicity, off‐target activation of other pathways and varying degrees of efficacy [71, 72, 73]. The comparative benefit of miR‐therapy is the wide range of target proteins and pathways that are dysregulated as a result of aberrant miRNA expression. While mTOR inhibition targets the mTOR pathway and downstream processes, miR‐126 is a significant haematopoietic regulator with numerous target proteins and pathways many of which are implicated in AML.

## 2 DISCUSSION

The delivery system employed in miRNA therapies is equally as important as the miRNA itself. Particularly in ensuring efficient delivery of the miRNA therapy to its target system and minimising toxicity. Meyer [74] commented on the potential issue of toxicity in reference to miR‐146a. However, this study utilised a CpG oligonucleotide‐conjugated delivery system and as demonstrated, the Tf‐NP delivery system displayed no negative impact on organ systems. The anionic formulation of the Tf‐NP system was further described by Huang, Schwind [75] as being specifically engineered to avoid nonspecific immune response, mitigate reticuloendothelial clearance and maximise disassociation of miRNA from the lipid complex upon endocytosis.

The most appropriate placement of miR‐therapies based on the current data would be as pre‐treatment specifically targeting LSCs and their capacity for self‐renewal to maximise CR. A recent study by Zeijlemaker et al. [76] highlighted the impact of LSC frequency on OS and CR with 49% more LSC‐free patients achieving 3‐year OS compared to patients with high LSC frequency, and a higher probability of achieving early CR. In patients without minimal residual disease (MRD), the presence of LSCs increased the 3‐year cumulative incidence of relapse by 18% and reduced 3‐year OS by 13%. Current strategies for LSC targeting in AML are not ideal, with antibody‐based treatments facing the hurdle of the complex molecular landscape of AML and the challenges of establishing reliable preferentially expressed targets and even available targets resulting in significant toxicity [77]. Of the six miR‐therapies detailed here, five had a significant impact on LSCs, CFUs and self‐renewal without altering normal HSCs (Table 2).

Another potential placement of miR‐therapy is as a sensitizing agent for relapsed/refractory AML. A number of miRNAs are implicated in chemoresistance, either directly or via their targets. A recent review by Fajardo‐Orduña et al. [78] commented on the issue of chemoresistance with >60% of AML patients developing relapsed/refractory disease. Their recommendations for combating this involve multi‐drug treatment, a response with potential increased toxicity and unknown efficacy in breaking resistance. While in contrast, miR‐181a has shown efficacy in sensitising AML cells to chemotherapy treatment with no reported additional toxicity [24]. Of the miRNA‐therapies discussed (Table 2), miR‐29b also demonstrated significant efficacy in improving therapeutic response.

Searches of the EU Clinical Trials Register and NIH: Clinical Trials.gov for miR‐21, miR‐29, miR‐126, miR‐181, miR‐196 and miR‐223 returned one result relevant to AML. The Phase I clinical trial was not primarily focused on miRNA (miR‐29b) treatment, but instead used increased miR‐29b as indicator of biological activity when assessing activity and tolerability of bortezomib/sorafenib/decitabine combination therapy, despite empirical evidence demonstrating the efficacy of these miRNAs in pre‐/post‐treatment and their lack of adverse effects.

## 3 SUMMARY

miRNAs are significantly dysregulated in AML and contribute to both disease progression and maintenance as well as treatment efficacy. Several of the miRNAs reviewed here have been shown to inhibit the key characteristics of AML in vitro or in vivo in model systems. However, to date, none of the miRNA therapies described have been developed for the treatment of AML. Greater efforts to develop miRNA therapies specifically for AML patients in the future may provide new treatment options and improve survival rates.

## AUTHOR CONTRIBUTIONS

D.F., E.B. and J.J. performed the research; B.G. designed the research study and secured funding; D.F. and J.J. analysed the literature and generated Tables; D.F. wrote the paper and D.F., P.U‐O. and B.G. edited the paper.

## COMPETING INTERESTS

The authors declare that they have no competing interests.
